# Editorial: Molecular Chaperones and Neurodegeneration

**DOI:** 10.3389/fnins.2017.00565

**Published:** 2017-10-11

**Authors:** Cintia Roodveldt, Tiago F. Outeiro, Janice E. A. Braun

**Affiliations:** ^1^Andalusian Center for Molecular Biology and Regenerative Medicine (CABIMER)-Spanish National Research Council (CSIC), Universidad Pablo de Olavide, University of Seville, Seville, Spain; ^2^Department of Experimental Neurodegeneration, Center for Nanoscale Microscopy and Molecular Physiology of the Brain, Center for Biostructural Imaging of Neurodegeneration, University Medical Center Göttingen, Göttingen, Germany; ^3^Hotchkiss Brain Institute, Department of Biochemistry and Molecular Biology, Cumming School of Medicine, University of Calgary, Calgary, AB, Canada

**Keywords:** heat-shock protein, molecular chaperone, proteostasis, amyloid protein, protein misfolding, neurodegenerative disease, neuroprotection, therapeutics

Molecular chaperones, including heat-shock proteins (HSPs), or stress proteins, are highly conserved proteins that play a critical role in the regulation of cellular protein homeostasis (proteostasis). Proteostasis is essential for the maintenance of the functionality of the proteome and, ultimately, of cells. Disruption of proteostasis leads to the accumulation of aberrantly folded proteins that typically lose their function. The accumulation of misfolded and aggregated proteins, due to genetic mutations, posttranslational modifications, or due to an age-related decline in cellular functions, can be also cytotoxic and has been linked to the pathogenesis of various degenerative diseases including those affecting the nervous system, such as Alzheimer's (AD), Parkinson's (PD) and Huntington's diseases (HD), or amyotrophic lateral sclerosis (ALS).

In addition to essential roles in *de novo* protein folding and the refolding of misfolded proteins, molecular chaperones are functionally diverse and participate in a myriad of cellular processes. These functions include preventing or resolving protein aggregation, and regulating proteostasis through fundamental processes such as the unfolded protein response (UPR), the heat shock response (HSR), chaperone-mediated autophagy (CMA), and the ubiquitin-proteasome system (UPS). Chaperones are important components of multiple cellular networks, as they form functional complexes with each other, with numerous co-chaperones regulating their function, and with hundreds of other cellular proteins. Therefore, they promote the crosstalk between various signaling pathways and regulate transcriptional networks. Finally, certain stress proteins display diverse roles in immunity. Given their ubiquitous cellular roles, the potentially common mechanisms of action that may apply, and the widespread consequences of their dysfunction, there is great interest in understanding how molecular chaperones function and how they may be manipulated to prevent or resolve protein aggregation linked to degenerative diseases, and particularly, in neurodegenerative disorders.

In this special issue, we have gathered 14 articles covering novel and significant aspects about the connection between chaperones and neurodegeneration, providing a series of updated and insightful views of the mechanisms and functions of a wide variety of molecular chaperones in the context of health and disease. Furthermore, this compilation offers a comprehensive overview of the most recent findings, advances, and implications as putative targets for therapeutic intervention. More specifically, we have arranged this special issue into three broad subjects, as follows: (A) General mechanisms of molecular chaperones in the maintenance of cellular proteostasis in health and disease; (B) Heat-shock proteins in aging, amyloid disease and cancer; (C) Functions and mechanisms of molecular chaperones within the nervous system.

## A. General mechanisms of molecular chaperones in the maintenance of cellular proteostasis in health and disease

Ciechanover and Kwon provide an updated general overview of the protein quality control mechanisms in the cell, with a focus on HSP functions in refolding and degradation of terminally misfolded proteins. Their review carefully covers the main protein degradation mechanisms, including the UPS, macroautophagy, and CMA. They also analyse our current understanding of the protective roles of chaperones and recent therapeutic studies in various neurodegenerative diseases. Lackie et al. focus on the HSP70/HSP90 HSPs, their structures, molecular mechanisms, and their cellular functions in combination with other chaperones, co-chaperones, and other protein partners, including the FKBP family members, the PPIase Cyp40, p23, and CHIP. Special attention is paid to the HSP90 co-chaperone Sti/Hop and its multiple roles in HSP90/HSP70-mediated functions in modulating protein misfolding, followed by an update on the main players and mechanisms of the chaperone machinery in the context of the synucleinopathies, HD, ALS, prion disease, and AD. On the other hand, Dubnikov and Cohen provide an insightful review on the emerging links between failure in early protein folding and maturation of secreted proteins and the development of neurodegerative disorders. They carefully analyse the early maturation events and the quality control mechanisms for terminally misfolded proteins within the ER, including ER-associated degradation (ERAD), the unfolded protein response (UPR), the formation of aggresomes, and other types of protein deposition sites, and their emerging association with neurodegenerative disease. Finally, Jackrel and Shorter describe our current knowledge and recent discoveries of molecular chaperones as protein-remodeling factors, to prevent or even reverse, protein aggregation processes in metazoans. In particular, they give a detailed overview of the recently characterized HSP110/HSP70/HSP40 disaggregase system, the NMNAT2 NAD-synthesizing enzyme, and the HtrA1 serine protease, from recent studies with misfolding disease models, while evaluating the therapeutic potential of yeast Hsp104 disaggregase for treating human misfolding diseases.

## B. Heat-shock proteins in the context of aging, amyloid disease and cancer

The review by Stroo et al. provides a comprehensive account of our current understanding of amyloid aggregates formation and its cellular regulators in health, disease and aging, including chaperone “disaggregases,” the role of chaperones in the main protein degradation pathways (UPS, macroautophagy, CMA), UPR, and protein compartmentalization. It also focuses on key aspects linking amyloidogenesis and neurodegeneration, such as amyloid formation modulators, the dysregulation of protein homeostasis processes, and the mechanisms of amyloid toxicity in neurodegenerative disease. The mini-review by Kuiper et al. focuses on polyglutamine (polyQ) protein aggregation associated to certain neurodegenerative disorders such as HD, as well as on the various factors affecting the aggregation process, particularly binding partners and molecular chaperones, e.g., CHIP and members of the DnaJ family. In addition to analyzing the multifactorial and complex nature of the process leading to disease initiation, the authors discuss the possibility that proper assessment of the different factors could help predict the age of onset of disease. Buxbaum and Johansson discuss the puzzling anti-amyloid activity of amyloidogenic transthyretin (TTR) protein and the BRICHOS domain on the AD-linked Aβ peptide, with a structural and mechanistic focus. The review also analyses the emerging links between TTR and BRICHOS-containing proteins and disease through amyloid formation, and also discusses potential therapeutic avenues for these amyloid precursors based on their anti-Aβ oligomerization properties. The review by Casas is a comprehensive and updated characterization of the ER stress-related chaperone GRP78 (also known as Bip), its multiple intracellular locations, interacting partners, and newly discovered functions including its key participation in ERAD, macroautophagy and the UPR. Furthermore, the author also elaborates a comparative analysis between the roles of Bip/GRP78 in tumor cytoprotection and neuroprotection in the context of neurodegenerative disease and aging. Finally, Calderwood and Murshid analyse the exacerbation or decline of HSP expression levels in cancer and AD, in the context of disease pathogenesis. In addition, their review provides an updated overview of the regulation mechanisms of the heat-shock response by HSF1 transcription factor, the key effector of HSP expression. The authors further discuss the emerging evidence of the HSF1-based dysregulation that might contribute to explain the intriguing negative epidemiologic correlation observed between cancer and AD.

## C. Functions and mechanisms of molecular chaperones within the nervous system

López-Ortega et al. report that long-term moderate reduction of the essential co-chaperone, cysteine string protein (CSPα/DnaJC5), reduces neuromusclar function. CSPα is essential for synapse maintenance and severe functional and structural changes occur in its absence. Through careful and detailed analysis, they demonstrate that 1 year old CSPα heterozygous mice, previously considered to be phenotypically normal, have impairment in neuromuscular function. Their findings imply that challenges lie ahead in identifying reduced levels of chaperones (like CSPα) that lead to mild synaptic impairment in patients. Gorenberg and Chandra provide an insightful review of four main players in synaptic proteostasis: CSPα/DnaJC5, auxilin/DnaJC6, RME-8/DnaJC13, and HSP110. Their review covers the unique features of proteostasis at the synapse highlighting both the current knowledge and current questions. Special attention is paid to the HSP110 disaggregase system and mutations in synaptic chaperones that cause human neurodegenerative diseases. On the other hand, Deane and Brown provide new information on HSPA6, a member of the HSPA chaperone family that is induced in neurons following heat shock. They carefully document the unique induction and localization of neural HSPA6 in comparison to two other HSPA family members, HSPA1A and HSP8. Given this differential targeting, they highlight the possible role of HSPA6 in human neurodegenerative disorders, emphasizing that while HSPA6 is present in the human genome it is absent from current mouse and rat models of neurodegenerative disease thereby creating a gap in our current understanding of HSPA6 function. Jung et al. provide an insightful review on the links between neurodegeneration and endoplasmic reticulum lipidostasis, proteostasis and calcium homeostasis. Special attention is paid to the role of calnexin, PDDIA3, BiP/GRP78 and cholesterol in the pathological sequence of events underlying neurodegeneration. Finally, Ousman et al. review the current knowledge of molecular chaperones following nerve injury, focusing on neuron survival, myelination, neuropathic pain, axon regeneration, and inflammation. They address how changes in molecular chaperone expression play an active role in injury or repair processes and highlight the therapeutic challenges involved in harnessing the beneficial properties while reducing the injurious functions of chaperones to enhance CNS and PNS recovery.

## Author contributions

CR was invited to prepare this Research Topic and invited TO and JB to be co-editors in it. We all prepared and discussed a list of guest authors, invited them, revised their manuscripts, and handled revisions performed by peer-reviewers.

### Conflict of interest statement

The authors declare that the research was conducted in the absence of any commercial or financial relationships that could be construed as a potential conflict of interest.

